# Vascular mimicry in zebrafish fin regeneration: how macrophages build new blood vessels

**DOI:** 10.1007/s10456-024-09914-y

**Published:** 2024-03-28

**Authors:** Anita Senk, Jennifer Fazzari, Valentin Djonov

**Affiliations:** Institute of Anatomy, University of Bern, Bern, Switzerland

**Keywords:** Vascular mimicry, Zebrafish, Macrophages, Regeneration

## Abstract

Vascular mimicry has been thoroughly investigated in tumor angiogenesis. In this study, we demonstrate for the first time that a process closely resembling tumor vascular mimicry is present during physiological blood vessel formation in tissue regeneration using the zebrafish fin regeneration assay. At the fin-regenerating front, vasculature is formed by mosaic blood vessels with endothelial-like cells possessing the morphological phenotype of a macrophage and co-expressing both endothelial and macrophage markers within single cells. Our data demonstrate that the vascular segments of the regenerating tissue expand, in part, through the transformation of adjacent macrophages into endothelial-like cells, forming functional, perfused channels and contributing to the de novo formation of microvasculature. Inhibiting the formation of tubular vascular-like structures by CVM-1118 prevents vascular mimicry and network formation resulting in a 70% shorter regeneration area with 60% reduced vessel growth and a complete absence of any signs of regeneration in half of the fin area. Additionally, this is associated with a significant reduction in macrophages. Furthermore, depleting macrophages using macrophage inhibitor PLX-3397, results in impaired tissue regeneration and blood vessel formation, namely a reduction in the regeneration area and vessel network by 75% in comparison to controls.

**Supplementary Information:**

The online version contains supplementary material available at 10.1007/s10456-024-09914-y.

## Introduction

Neoangiogenesis is an essential adaptive process during many physiological and pathological processes resulting in the formation of new microvascular segments [1–8]. Over the past years, three major angiogenic mechanisms (sprouting angiogenesis, splitting (intussusceptive) angiogenesis and vascular mimicry) have been well investigated [9, 10]. Vascular mimicry (VM) is characterized by the formation of matrix-rich, vasculogenic-like channels containing aggressive tumor cells that transdifferentiate into multiple cellular phenotypes and obtain endothelial-like characteristics. Such channels are not true blood vessels, but mimic their function by building vascular-like structures that contain plasma and red blood cells, ultimately contributing to the de novo formation of the microvasculature [11–13]. Such vascular networks support blood flow to the tumor thereby promoting tumor progression and poor clinical outcomes [10, 14–17]. The VM mechanism is completely independent of endothelial cell (EC) proliferation and migration [11, 18]. Although the initial description of VM was challenged [19], it has been subsequently observed in several malignant tumors such as melanoma [11], breast cancer [20], prostate cancer [21], hepatocellular carcinoma [22], gastric cancer [23], ovarian cancer [18] and lung cancer [24]. Recent findings suggest that VM can also be involved in tumor metastasis [25] and the development of resistance to angiostatic compounds [26] as these vascular-like structures formed by VM-positive tumor cells are not sensitive to classical inhibitors of angiogenesis [17, 27, 28]. Furthermore, angiostatic treatment has even been shown to induce VM development [29]. Thus, a better understanding of VM mechanisms is essential for providing new therapeutic opportunities with better treatment prognosis.

Due to the optical transparency, high number of offspring, possibility of genetic manipulations, ease of maintenance, rapid development and amazing capability for tissue regeneration, the zebrafish model has become commonly used in the field of angiogenesis and tissue regeneration [30]. Although the zebrafish seems far removed from humans, they share remarkable similarities at the molecular, physiological and anatomical level [31]. Transgenic zebrafish lines, having fluorescent proteins expressed under specific tissue promotors, enabling the investigation into the proliferative and migratory behaviors of cell types involved in new blood vessel formation in vivo [30]. In addition, the zebrafish has the potential to completely regenerate many of its tissues, such as the heart, retina, spinal cord, scales and fins [32]. In particular, the caudal fin provides an ideal tissue for investigating vascular regeneration in adult zebrafish due to its simple, thin architecture, relative transparency, accessibility and almost unlimited regenerative capacity [33]. The caudal fin contains segmented bony rays and inter-ray mesenchymal tissue, filled with blood vessels and nerve axons [32]. It has been shown that the adult caudal fin is regenerated within 30 days and depends on appropriate wound healing processes such as the creation of blastemal progenitor cells and on their progressive redifferentiation [34–36].

Macrophages (MΦs) are a heterogeneous population of cells which play different roles during immune responses, tissue regeneration and sprouting angiogenesis [37, 38]. It is well known that MΦs are essential for caudal fin regeneration. During the early phases of inflammation, soon after fin amputation, MΦs are recruited to the wound, where they function as phagocytic cells. Then, in late stages, MΦs support proper blastemal formation [39, 40]. In addition, it has been documented that MΦs could act as cellular chaperones to fill the gap between ECs in damaged endothelium [41]. Furthermore, MΦs also secrete angiogenic growth factors and thus support sprouting angiogenesis [42]. Most importantly, It has been shown that MΦs also express some endothelial markers [43], which suggest that MΦs could transdifferentiate into ECs. Although the general importance of MΦs supporting tissue regeneration and sprouting angiogenesis is well known, the role of MΦs in VM processes during tissue regeneration is less clear.

VM was first described in 1999 [11] and is now one of the most intensively investigated phenomenon in tumor angiogenesis [44, 45]. As mentioned, this process has been documented in many different cancer types, but to our knowledge has never been reported as a part of normal, physiological blood vessel formation. Therefore, we used this model to investigate whether vascular mimicry also occurs during the regeneration of normal tissue and if so, what are the main cell types involved. In the present study, we demonstrate that a process closely resembling tumor vascular mimicry is, in fact, involved in physiological blood vessel formation during tissue regeneration.

## Materials and methods

### Animal care and maintenance

For the entire study, adult transgenic zebrafish, aged 10–20 months, were used. Zebrafish were reared and kept in the water system (Tecniplast ZebTEC facility) at the temperature of 28.5 °C, conductivity of 500µS and maintaining a pH level of 7.4. The fish followed a 14:10 h light and dark diurnal rhythm. They were nourished twice a day with a combination of live brine shrimp (Special Diets Services, Germany) and dry food (GM-300, Skretting, France). The following transgenic zebrafish lines were used: Tg(fli1a:EGFP)//Tg(mpeg:mCherry) in which ECs express a green fluorescent protein [46] and macrophages express a red fluorescent protein [47]; Tg(fli1a:EGFP) in which ECs are labelled with a green fluorescent protein [46] and wild type (AB) zebrafish line (ZFIN Germany), which has no fluorescent expression. All animal experiments were performed according to the ARRIVE [48] guidelines, the guidelines of the Swiss Animal Welfare Act, and approved by the Veterinary Office of the Canton Bern and Swiss Animal Welfare Ordinance. The study protocol was endorsed by the Bernese Cantonal Animal Welfare Commission and was supported with the corresponding permit issued by the Bernese Cantonal Veterinary Office (Nr. BE67/18). All procedures were carried out under anesthesia with Tricaine (MS-222, Sigma-Aldrich), and all efforts were made to minimize suffering. On a daily basis, the zebrafish were observed for their swimming behavior, estimated body weight (visually assessed), and overall well-being. Throughout the duration of the study, no indications of pain or distress were noted in any of the zebrafish.

### Fin amputation

Fin amputation was performed as previously described by Senk and Djonov [49]. Briefly, the amputation of caudal fin (approx. 50% lesion size) was accomplished under anesthesia using 0.04% Tricaine perpendicular to the cranio-caudal axis of a zebrafish using a razor blade. Following the amputation procedure, the zebrafish recovered and were subsequently returned to the same water system.

### In vivo imaging

The process of caudal fin regeneration was monitored at several time points up to 15 days post amputation, as previously described by Senk and Djonov [49]. At each time point, zebrafish were anaesthetized with 0.04% Tricaine, transferred to the petri dish where they were immobilized with thin cover glass. Data were obtained by Leica M205FA stereomicroscope; high-resolution in vivo imaging was performed with the Zeiss LSM880, using a 20× air objective lens. Following the imaging process, all fish recovered and were returned to the same water system. Data were visualized and analyzed using ImageJ software.

### Transmission electron microscopy (TEM)

Amputated fins were processed according to a standard protocol as previously described [49]. Briefly, the fins were fixed in Karnovsky’s solution overnight at 4 °C, rinsed in sodium cacodylate buffer, dehydrated through a series of ethanol concentrations and embedded in EPON resin (Sigma-Aldrich). Subsequently, blocks were sliced into 60 nm-thick sections using an ultra-microtome (Leica) equipped with a diamond knife (Diatome). Sections were placed on copper specimen grids (Plano) and stained with uranyl acetate and lead citrate for 40 min. The specimens were examined using a Philips TEM CM12 electron microscope.

### Flow cytometry

Adult zebrafish caudal fins were amputated and dissociated by vigorous shaking for 1 h at room temperature (RT) in a solution of Liberase DH Research Grade (Roche) reconstituted in Hank’s Buffered Salt Solution (HBSS) with gentle pipetting up and down. The solution was then centrifuged at 450 xg for 5 min at 4 °C and supernatant discarded. Cell pellets were re-suspended in 1xPBS containing a viability marker (LIVE/DEAD Fixable Violet Dead Cell Stain Kit, 405 nm (Invitrogen)) and the suspension was incubated for 30 min at RT, washed in 1x PBS, centrifuged at 450 xg for 5 min at 4 °C and supernatant discarded. Finally, for cell cytometry, cell pellets were re-suspended in 500 µl of FC buffer and run on a BD LSR II Special Order System (SORP) and subsequently analyzed with FlowJo V10 Software. Negative control (wild type zebrafish line) and samples (Tg(fli1a:EGFP)//Tg(mpeg:mCherry)) were processed and analyzed at six time points (at 0dpa - reference, 1dpa, 3dpa, 5dpa, 7dpa and 10dpa; *n* = 7).

### Whole-mount immunofluorescence staining and imaging

Caudal fins were collected, fixed with 4% paraformaldehyde in 1xPBS for 6 h at + 4°C, washed three times in 1xPBS and stored in 70% ethanol until immunofluorescence (IF) staining was performed. IF was performed using the following sets of primary antibodies: Rat monoclonal anti-mCherry (ab2536611, Thermo Fisher Scientific) diluted 1:250; and Chicken polyclonal anti-GFP (ab2307313, aveslab) diluted 1:250. Secondary antibodies were the following: Goat anti-rat IgG Alexa Fluor 555 (ab150158, abcam) diluted 1:250; goat anti-chicken IgG Alexa Fluor 488 (ab150169, abcam) diluted 1:500. IF staining was performed using the following protocol: Tissue permeabilization with 0.3% Triton X-100 (Sigma-Aldrich) in 1xPBS for 10–15 min; followed by blocking for 1 h at RT with blocking solution (10% goat serum, 3% milk, 0.3% Triton X-100 in 1xPBS) followed by overnight incubation with primary antibody at 4°C. Three washes of 10 min each with 0.1% Triton X-100 in 1xPBS were performed after incubation and the secondary antibody was diluted together with 4’,6-Diamidino-2-phenylindole dihydrochloride (DAPI; 1:1000) and incubated for 5-6 h at 4 °C. After several washes with 0.1% Triton X-100 in 1xPBS, fins were mounted with ProLong Glass Hard-Set Antifade Mountant (Thermo Fisher Scientific) and stored at 4 °C until imagining. Images were acquired by a Zeiss 780 confocal microscope fitted with a 20× objective 1.0 NA with a dipping lens.

### Pharmacologic treatments

Following the caudal fin amputation, fish were incubated in a water system containing the following compounds: CVM-1118 (125nM) and Pexidartinib (PLX-3397; 500nM)).

#### CVM-1118

CVM-1118 is a small molecular compound, currently under clinical development for anti-cancer treatment with particular interest in targeting VM. It has been reported, that CVM-1118 treatment results in decreased cell proliferation and increased apoptosis. Moreover, a breakdown in the cell’s ability to form branching, tubular networks characteristic of VM has been documented [10]. Before conducting the main experiment, an initial drug optimization study was performed, in which five different concentrations of CVM-1118 were tested. Based on the fish phenotype and welfare, we decided to use a concentration of 125 nM CVM-1118 dissolved in normal fish water with an exposure period of 15 days. Briefly, at day 0, caudal fin amputation was performed and fish were placed in 2 tanks – one containing normal fish water (control group) and a second containing 125nM CV-1118 (treated group). Five fish per group were investigated at six time points. The fish had their regular diet and were kept in the same tanks throughout the entire duration of the experiment.

#### Pexidartinib (PLX-3397)

PLX-3397 is selective ATP-competitive colony stimulating factor 1 receptor (CSF1R or M-CSFR) inhibitor. CSF1 regulates the proliferation, differentiation, and survival of macrophages [50], which means that PLX-3397 systematically depletes macrophages that has been already shown in the literature [50–52]. Prior to the actual experiment, an initial drug optimization experiment was performed, in which five different concentrations of PLX-3397 (Plexxikon Inc., Berkeley, CA, USA) dissolved in 0.05% dimethyl sulfoxide in water were tested. Based on the fish phenotype and welfare, we decided to use a concentration of 500nM PLX-3397 with an exposure period of 15 days. Briefly, fish were immersed in a drug solution 3 days before the performed amputation and maintained under those conditions during the entire course of the experiment. The PLX-3397 solution was changed every second day. Fish in a PLX-3397 threated group were compared to those having fin amputation and being placed in normal fish water (control group). Five fish per group were investigated at six time points and were fed normally during the entire experiment.

### Quantification of fin regeneration

The zebrafish caudal fin is considered a 2D-structure consisting of the supplying artery and two veins per ray with connecting capillaries [53]. Two variables describing the whole zebrafish caudal fin were introduced, namely, “total regenerated area” (TRA) and “vascular projection area” (VPA). TRA refers to the area of the regenerated caudal fin, irrespective of its vascularization. On the other hand, VPA represents the cumulative area of all vessels within the regenerated part, as visualized on the fluorescence images [34].

### Statistical analysis

Data was analyzed using one-way ANOVA and multiple t-tests in GraphPad Prism v8. A p-value of less than 0.05 was considered statistically significant and marked by asterisks (*p < 0.05; **p < 0.01; ***p < 0.001 and ****p < 0.0001; “n” represents the number of biological replicates.

## Results

### Mosaic blood vessels within the regenerating zebrafish caudal fin

**Fig. 1:**
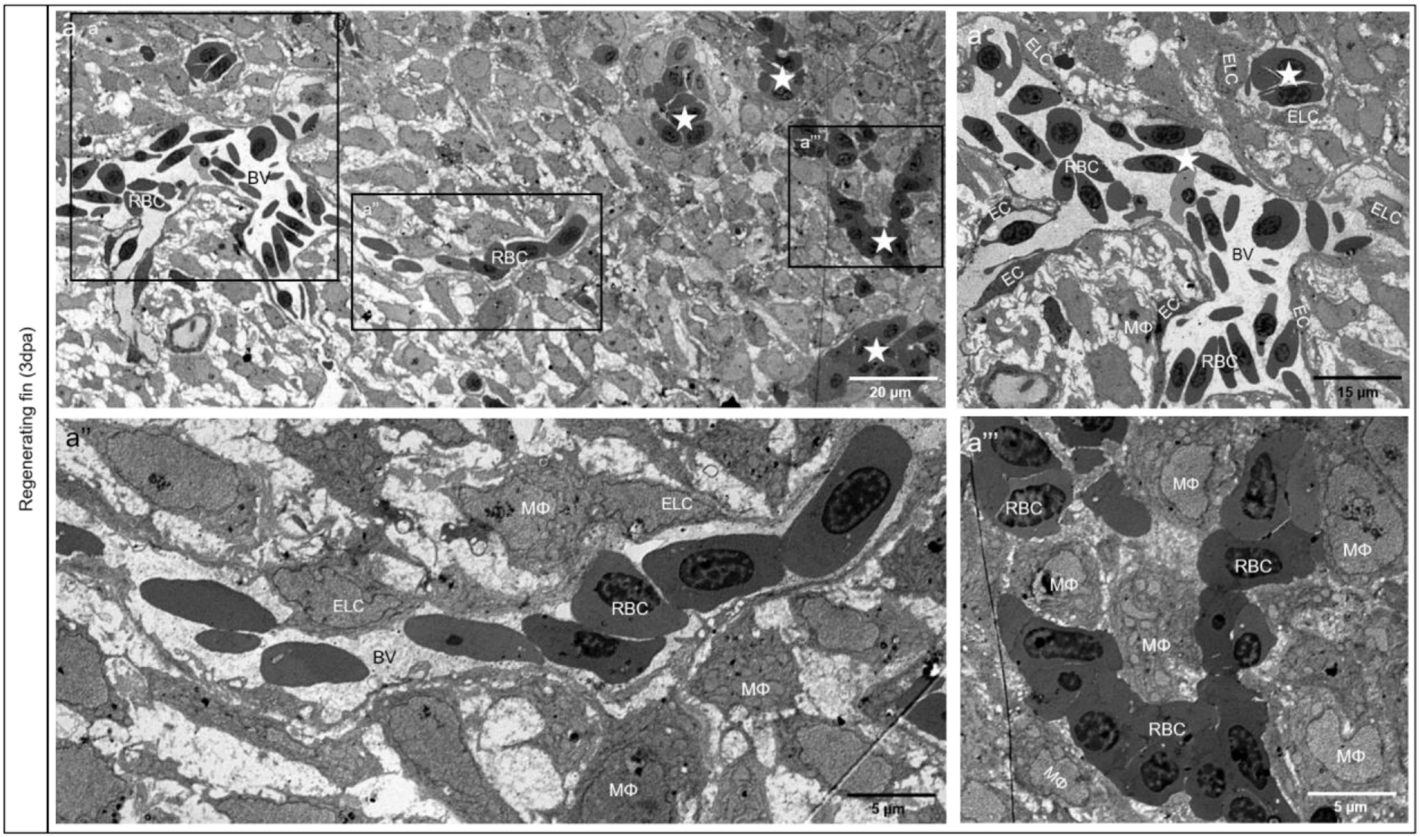
Endothelial cell mosaic during neoangiogenesis in the regenerating zebrafish fin. (**a**) an overview of the regenerative tissue at 3dpa with perfused blood vessels (BV) containing red blood cells (RBC), and extravasal clusters (asterisks) or single RBC; the distal part of the fin is depicted on the right side. The proximal part of the fin with large and perfused blood vessel with well-differentiated endothelial cells (EC) and classical morphological appearance is depicted in **a**’. At the distal (right) part, first vessels containing a mosaic of typical EC and Endothelial-Like Cells (ELC) are detectible (asterisks). **a**’’ depicts a transient segment with continuous vascular coverage. The latter consist of ELCs with long sleeves surrounding the RBCs. The morphological appearance of the ELCs closely resembles the macrophage (MΦ) structural phenotype. Different, transient forms are detectible. At the vascular front, the cells surrounding the RBC clusters are classical MΦ (**a**’’’). Images are acquired by transmission electron microscopy

**Fig. 2:**
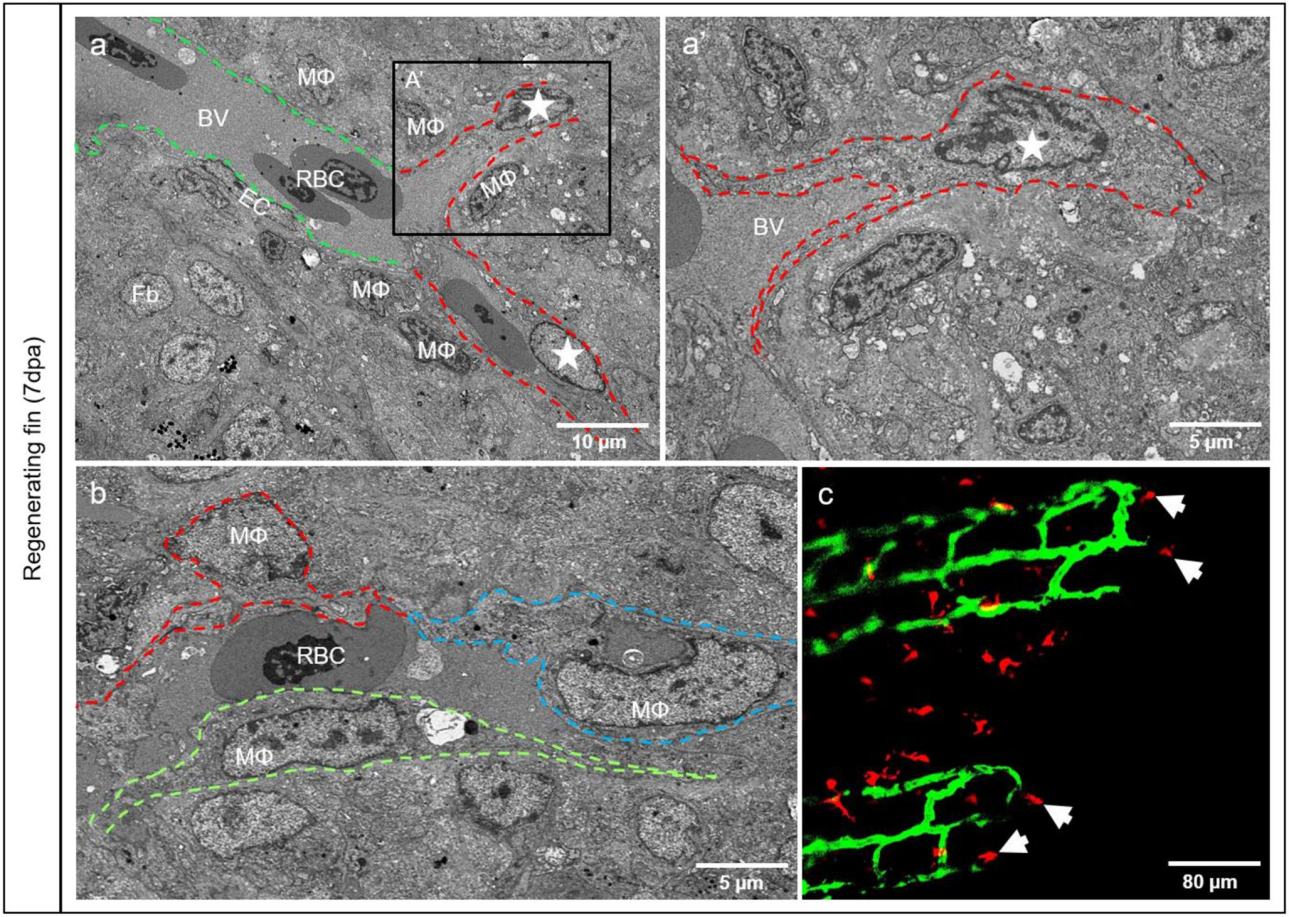
The vasculature expands by transformation of the adjacent macrophages into endothelial like cells. **a**) mosaic blood vessel containing a typical blood vessel segment with well-defined ECs (green dash line), and an atypical one appearing as a channel (red dash line) covered by MΦ (asterisk) extensions. At higher magnification (**a**’), MΦ at the vascular tip (asterisk) send a cytoplasmic extensions (dotted line) to the stock EC cells. (**b**) elongated MΦ (traced red, blue and green) is part of the vascular wall with extensions fencing the lumen containing plasma and RBCs. In vivo investigations (ECs appear in green and MΦ in red) confirmed MΦ at the front of the vessel tips (**c**, arrows) corresponding to that demonstrated in a and **a**’ by TEM. Images **a**, **a**’ and **b** are acquired by the transmission electron microscope and image c by confocal microscopy

A morphological overview of the regenerating tissue was documented showing perfused blood vessels as indicated by multiple red blood cells (RBCs) as well as extravascular RBC clusters or single RBCs at 3dpa (Fig. 1). The proximal part of the fin contains large, perfused blood vessels with well-differentiated endothelial cells (ECs) (Fig. 1a’). In distal fin segments, mosaic blood vessels accommodate typical ECs and Endothelial-Like Cells (ELCs). ELCs are rounded and extend long sleeves around the vessel lumen (Fig. 1a’’). Morphologically, ELCs closely resemble the structural phenotype of macrophages (MΦ). Additionally, many classical MΦs near ELCs are detectible (Fig. 1a’’). At the vascular front, RBC clusters are surrounded with multiple MΦs (Fig. 1a’’’). Later, at 7dpa, mosaic blood vessels contain a typical blood vessel with well-defined ECs (green dash line) filled with RBCs and atypical ones appearing as a channel (red dash line) with MΦ at the tip (Fig. 2a). MΦs appear to elongate into the blood vessel wall (Fig. 2a’ and Fig. 2b) by sending projections towards the lumen containing plasma and RBCs. In vivo observations reveal MΦs, in red (Fig. 2c, arrows), are actively interacting and making contacts with the ECs in green at the front of the vessel tips (Fig. 2c). Additionally, these MΦs (Video 1, white arrows) are moving towards and away from ECs (green); it seems that they are “drilling” space in front of the sprouting vessels.

### Macrophages contribute to blood vessel network formation during fin regeneration

**Fig. 3:**
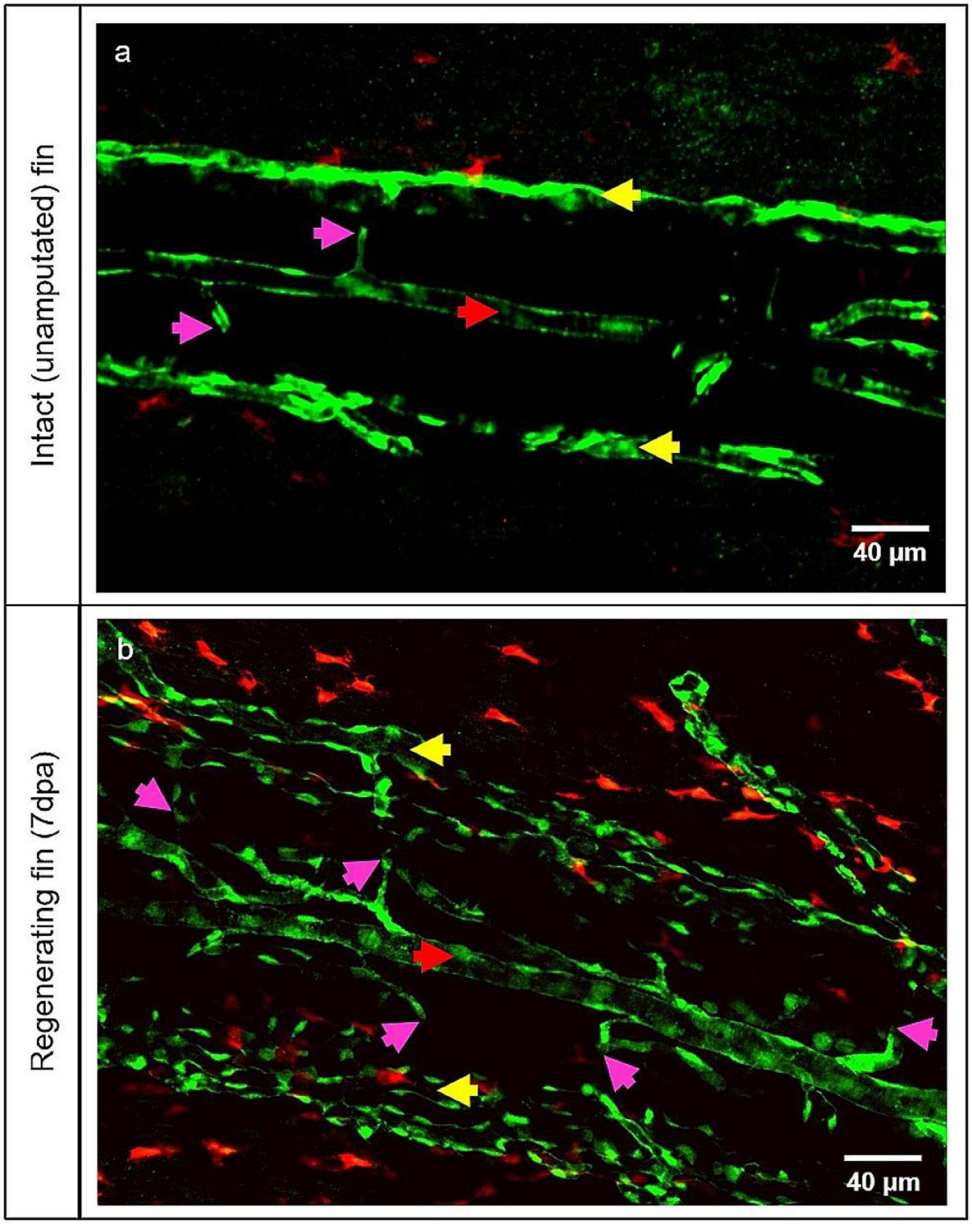
MΦ augmentation during the caudal fin regeneration. In vivo appearance of MΦ by the intact (unamputated) fin (**a**) and regenerating fin at 7dpa (**b**). Blood vessels (ECs) appear in green and MΦ in red (transgenic line). (**a**) displays hierarchically simple-organized blood vessels containing an artery (red arrow), veins (yellow arrows) and connecting capillaries (purple arrows), and only a few tissue MΦ. (**b**) displays a dense, complex blood vessel network with multiple MΦ surrounding the blood vessels and scattered in the tissue in between. Right side – zebrafish tail; images acquired by confocal microscopy

Prior to investigating the presence of the double positive cells detected by the IF staining and confocal microscopy, the difference between the intact (unamputated) and regenerating fin at 7dpa was first examined focusing on the appearance of MΦ (Fig. 3). In the intact fin, few MΦs were detected (Fig. 3a). However, upon amputation (at 7dpa), multiple MΦs invaded the blood vessels and the tissue between vessels.

**Fig. 4:**
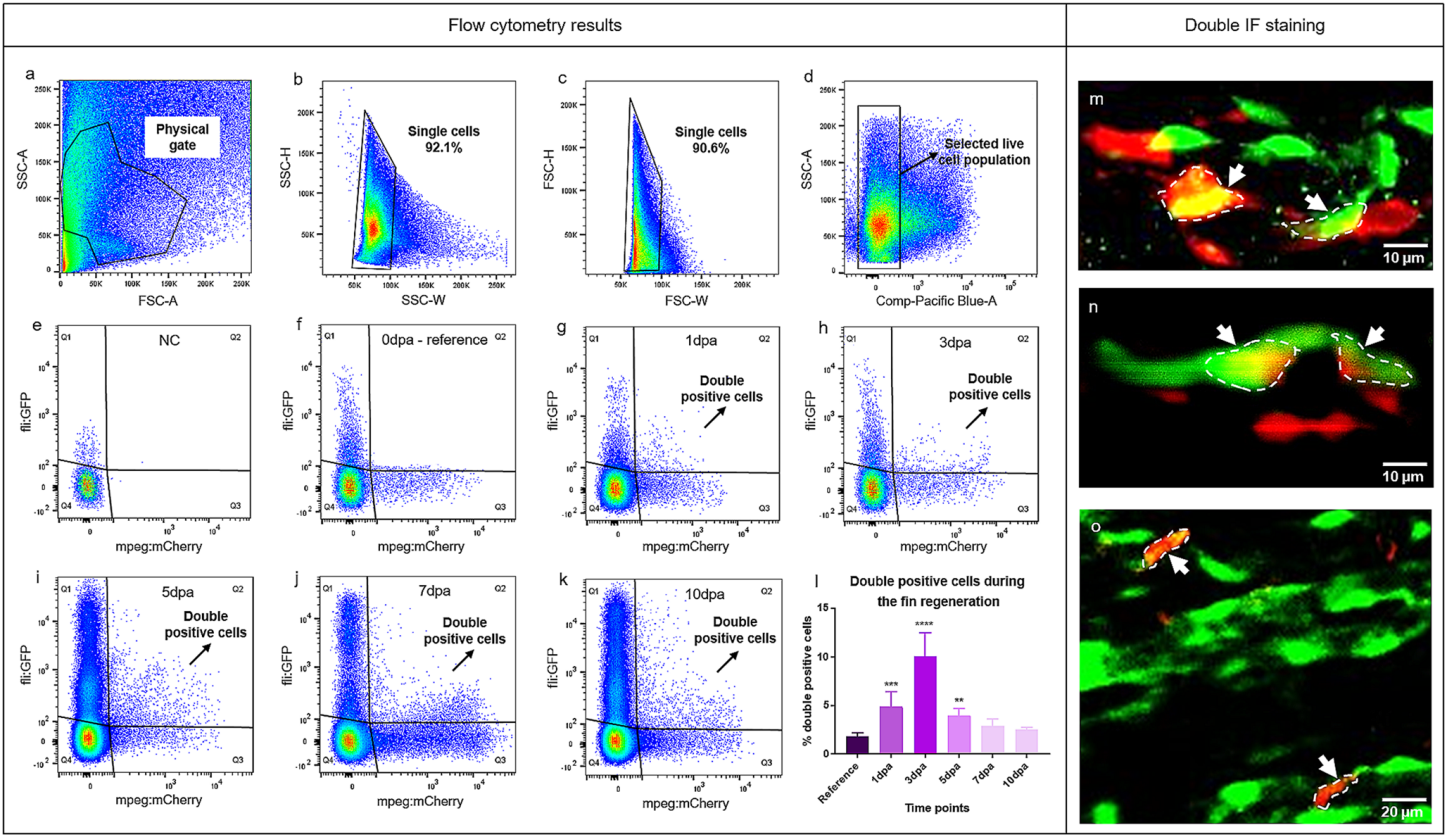
Co-localization of MΦ and EC markers at the regenerating vascular front. Plots (**a**-**k**) and quantification of double positive cells (**l**) from flow cytometry analysis are shown. Plots a, b, c and d represent gating parameters and selection of targeted cell population. Plot **e **shows the negative control (NC);** f-k** display representative plots during the early caudal fin regeneration (0dpa, 1dpa, 3dpa, 5dpa, 7dpa, 10dpa). Q1 represents green positive cells (ECs), Q2 represents double positive cells expressing MΦ and EC markers in a single cell, Q3 represents red positive cells (MΦ) and Q4 represents double negative cells. Graph l displays quantification of double positive cells over the course of 10 days (0dpa-10dpa, *n* = 7). Percentage of double positive cells significantly increased at 1dpa, 3dpa and 5dpa. Double whole-mount immunofluorescence staining is showing ECs in green and MΦ in red in the regenerating fin at 7dpa (**m**-**o**). m-o reveals the overlap of EC and MΦ markers within a single cell (white dash line, arrows). Images are acquired by confocal microscopy

Data obtained from flow cytometry analysis revealed EC and MΦ marker co-localization at the regenerating front at six time points (0dpa, 1dpa, 3dpa, 5dpa, 7dpa, 10dpa) (Fig. 4). Double positive cells, expressing both EC (fli1a) and MΦ (mpeg) markers, appeared between 1dpa and 10dpa (Fig. 4g and k); and increased significantly between 1dpa and 5dpa (Fig. 4l). Double positive cells (in %) reached their peak at 3dpa and their amount increased by approximately 80% in comparison to the reference (0dpa). The percentage of double positive cells remained above baseline levels at 7dpa and 10dpa, but the difference was not statistically significant (Fig. 4l).

To visualize the presence of double positive cells at the regeneration front, immunofluorescence (IF) staining was performed (Fig. 4m and o). Co-localization of ECs in green and MΦs in red was confirmed by double whole-mount immunofluorescence (IF) staining. IF demonstrated cells that were positive for both EC and MΦ markers (Fig. 4m and o, arrow and dash line).

### Effect of vascular mimicry inhibitor CVM-1118 on regenerative angiogenesis in zebrafish caudal fin

**Fig. 5:**
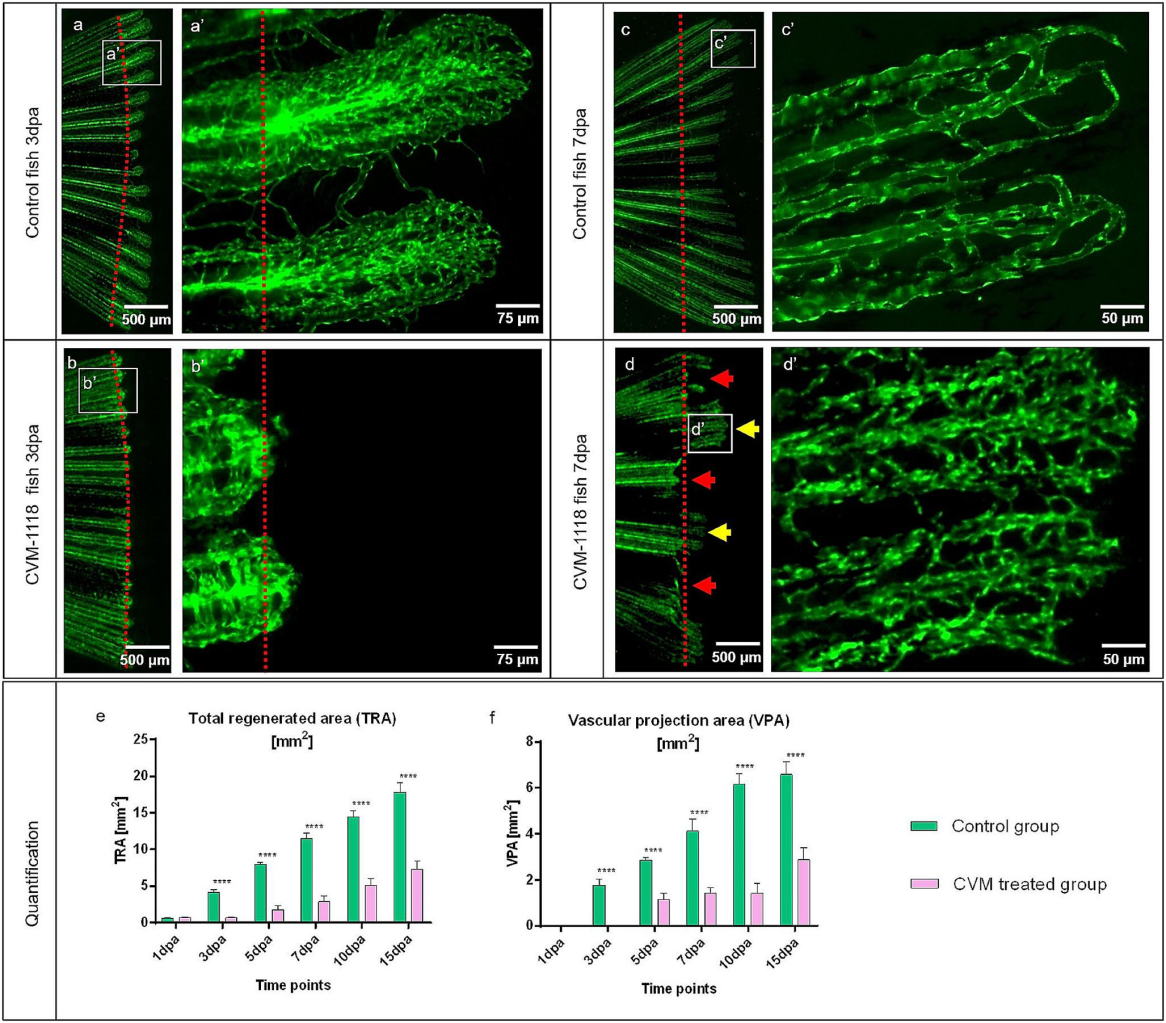
Inhibition of vascular mimicry by CVM-1118 impairs caudal fin regeneration and blood vessel formation. Vascular changes during normal fin regeneration (**a**, **c**) versus regeneration in the presence of CVM-1118-treated group (**b**, **d**) at 3dpa and 7dpa (green, transgenic zebrafish line; red dash line – amputation plane). Blood vessels of the controls (**a**, **a**’ and **c**, **c**’) appear well organized and represent the classical vascular regeneration pattern. **b** and **b**’ display shorter regeneration area with respectively modest vascularization in treated animals; hierarchical vessels and capillaries are less pronounced. At 7dpa, the regenerating tissue is severely impaired; smaller vascular plexus and regeneration processes, including blood vessel formation, is completely absent in half of the fin area (**d**, red arrows) in comparison to the control. In the partially regenerated regions (**d**, yellow arrows), tissue and the vascular plexus appear shorter with a dense and poorly organized capillary meshwork (**d**’). Images are acquired by fluorescent reflected light microscopy. Quantification of the tissue regeneration and vascularization after inhibition was assessed by two variables: Total regenerated area (TRA = regenerated fin in mm2; (**e**) and vascular projection area (VPA = vessels within regenerated part in mm2; **f**) during the period of 15 days in control group (green) versus CVM-1118 treated group (purpura); *n* = 5. In the treated group, TRA and VPA are significantly smaller (about 70%); fin shape and size never returned to the amputated stages indicated by control

Differences in tissue regeneration and vessel formation between the control group and CVM-1118-treated group during early caudal fin regeneration has been documented at 3dpa and 7dpa (Fig. 5). In both control groups (3dpa and 7dpa), the regenerating tissue contains a well-organized blood vessel network (Fig. 5a’, 5c’). In the CVM-1118-treated group (3dpa), the regenerated area and the vascular plexus appear shorter. Vascularization is modest, and vessels and capillaries are less pronounced (Fig. 5b and b’) compared to the untreated group (Fig. 5a and a’). Later, at 7dpa, in CVM-1118-treated animals, the regenerating region is severely impaired with a smaller vascular plexus and the absence of characteristics of the regeneration process including blood vessel formation in half of the fin area (Fig. 5d, red arrows) relative to the control (Fig. 5c). In addition, the tissue itself and its respective vascular plexus appear shorter with dense capillary meshwork with very tiny (less than 4–5 μm) unperfused capillary segments (Fig. 5d’).

**Fig. 6:**
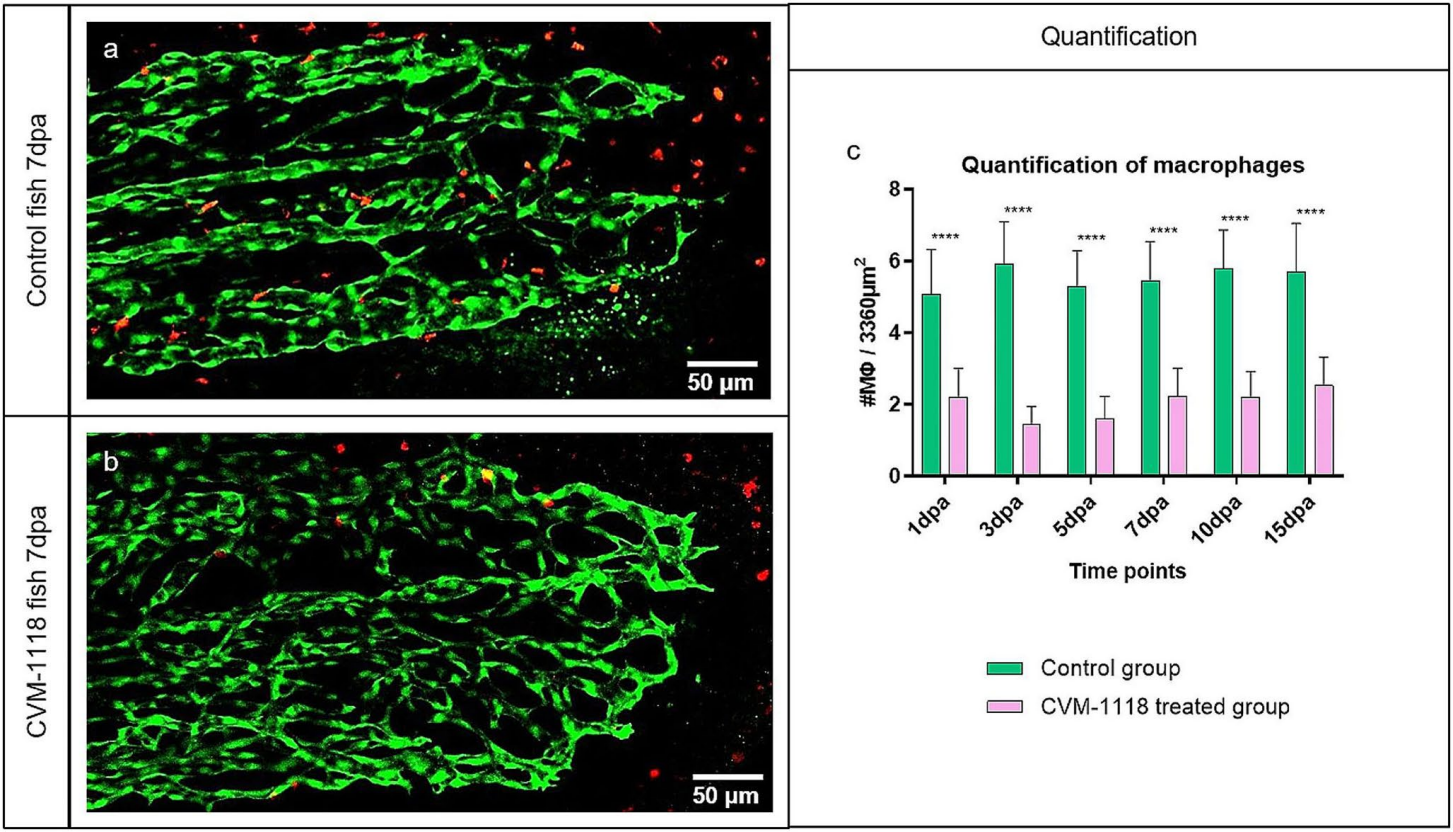
Vascular mimicry inhibitor CVM-1118 causes macrophage reduction in the regenerating fin. MΦ appearance during normal fin regeneration (**a**) versus CVM-1118-treated group (**b**) at 7dpa and their quantification during the period of 15 days (**c**); *n* = 100. In the treated group, MΦs surrounding the blood vessels and their presence at the front of the vessel tips appear lacking (**b**); the amount of MΦ is significantly reduced (about 60%) for the entire course of the experiment (**c**), which indicates that the vascular mimicry inhibitor CVM-1118 has a cytotoxic effect on MΦs as well. Images are acquired by confocal microscopy; green – ECs, red – MΦs (transgenic zebrafish line)

The effect of the vascular mimicry inhibitor CVM-1118 on regenerative angiogenesis was quantified by two variables: The total regenerated area (TRA = regenerated fin in mm2) and vascular projection area (VPA = vessels growth within regenerated fin in mm2) (Fig. 5e and f). TRA in the CVM-1118-treated group from 3dpa is significantly reduced relative to control (*p* < 0.0001) and fin size never reached pre-amputation levels as observed in control animals (Fig. 5e). Vessel growth was not observed at 1dpa. After the formation of the vascular plexus, a rapid increase in VPA has been documented in the control group at 3dpa and in the CVM-1118-treated group at 5dpa (Fig. 5f). In the treated group, regeneration and vessel growth were reduced by approximately 70% for TRA (Fig. 5e) and 60% for VPA (Fig. 5f), never reaching those of the controls. Taken together, treatment with the vascular mimicry inhibitor (CVM-1118) significantly impairs tissue regeneration by obstructing vessel formation and expansion. In addition, CVM-1118 reduced the presence of macrophages in the regenerated fin (Fig. 6). Upon the treatment, the amount of MΦs is greatly decreased by approximately 60% (Fig. 6c) and the MΦs around the blood vessels and their presence at the front of the vessel tips is lacking (Fig. 6b).

### Caudal fin regeneration and blood vessel formation are affected by the macrophage inhibitor PLX-3397

**Fig. 7:**
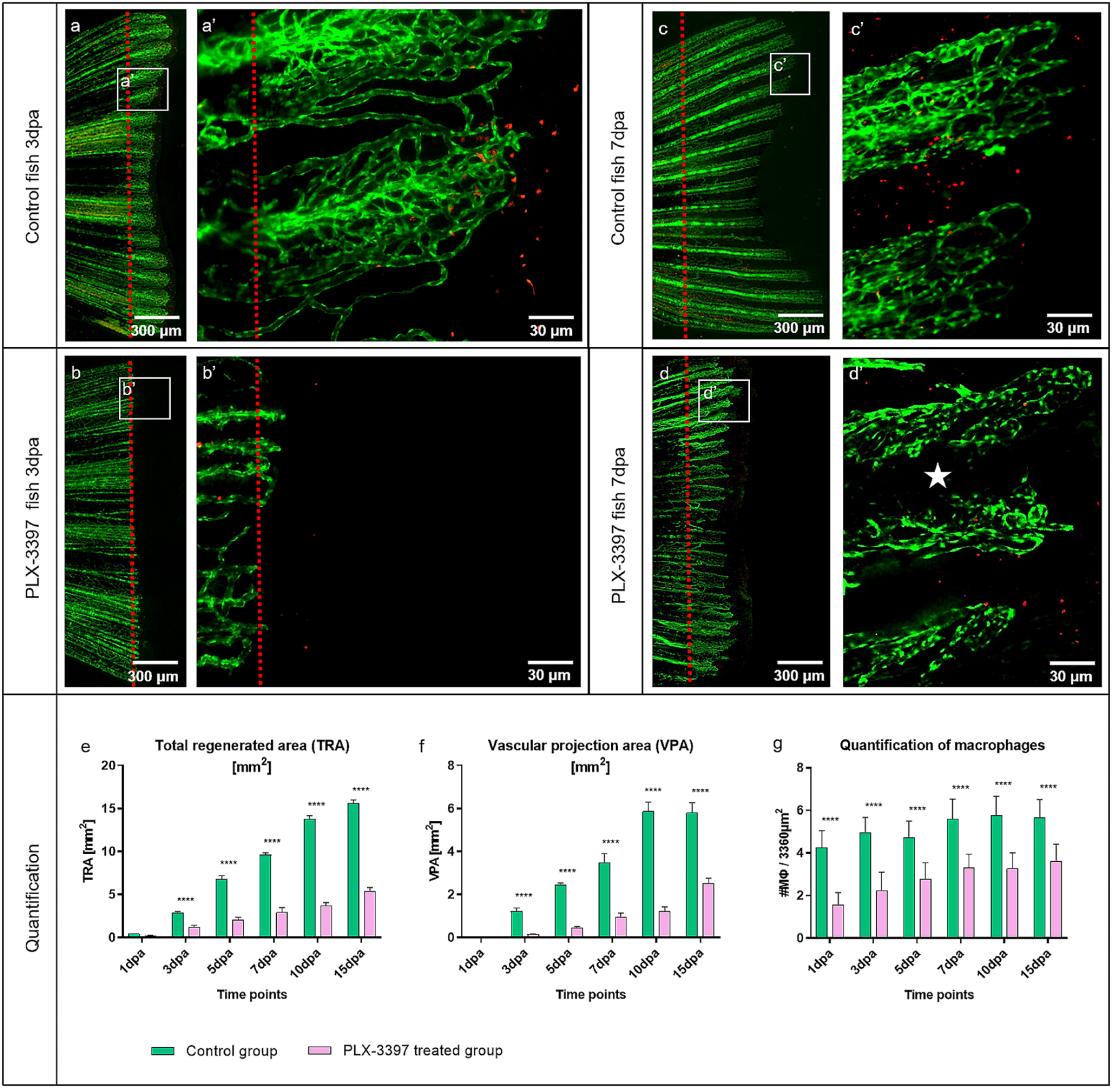
Macrophage inhibitor PLX-3397 impairs caudal fin regeneration, blood vessel formation and macrophage appearance. Vascular alteration during normal fin regeneration (**a**, **c**) versus PLX-3397-treated group (**b**, **d**) at 3dpa and 7dpa is documented (green – ECs, red – MΦs; transgenic zebrafish line; red dash line – amputation plane). In the control animals, classical tissue regeneration pattern, blood vessel morphology and MΦs are documented (**a**, **c**). Regenerative area and vascular plexus is severely impaired and underdeveloped in the PLX-3397-treated group (**b**, **b**’) at 3dpa; only a few capillaries are observed and less MΦs detectible. In the treated group, at 7dpa, the regenerating region and the vascular plexus are smaller, modest and capillaries are less pronounced (**d**, **d**’). Intraray region is not properly vascularized (d’, white asterisk) and less MΦs are observed in comparison to the control group. Images are acquired by fluorescent reflected light and confocal microscopy. Quantification of the regeneration and vascularization after the MΦ elimination has been performed by two variables: total regenerated area (TRA = regenerated fin in mm2; (**e**), vascular projection area (VPA = vessels growth within regenerated fin in mm2 (**f**)) during the period of 15 days in control group (green) versus PLX-3397-treated group (purpura). *n* = 5. (**h**) Quantification of MΦ amount between control and PLX-3397-treated animals; *n* = 100. In the treated group, TRA and VPA are significantly smaller (about 75%), never reaching the parameters of the controls (**e**, **f**); Amount of MΦ is significantly reduced (about 50%) in the PLX-3397-tretaed animals during the entire course of experiment in comparison to control animals (**g**)

Caudal fin regeneration and vascular changes between control group and PLX-3397 treated group at 3dpa and 7dpa has been documented (Fig. 7). In the control animals, standard tissue regeneration containing a well-organized blood vessel network and MΦ appearance was observed (Fig. 7a and a’, 7c, 7c’). Upon PLX-3397 treatment, tissue regeneration was severely impaired and regenerated fins were shorter in comparison to controls (Fig. 7b and d). Blood vessel formation was minimal and those that were observed were immature, underdeveloped, tiny and unperfused. Furthermore, MΦ density was reduced (Fig. 7b’, 7d’). In the treated animals, at 7dpa, the intraray region is not properly vascularized (Fig. 7d’, white asterisk) and capillaries were modest and less pronounced.

The effect of the macrophage inhibitor PLX-3397 on regenerative angiogenesis was quantified by two variables: the total regenerated area (TRA = regenerated fin in mm2) and vascular projection area (VPA = vessels growth within regenerated fin in mm2) (Fig. 7e and f). In the treated group, TRA and VPA were significantly smaller (about 75%) and never reached the parameters of the controls. The quantity of MΦs is also dramatically reduced (about 50%) in the PLX-3397 treated animals for the entire course of the experiment in comparison to control animals (Fig. 7g).

## Discussion

VM is a recently discovered angiogenetic process found in many malignant tumors in which vessels are formed independently of vascular endothelium as in the typical angiogenic process. In contrast, it involves the formation of vascular structures composed of tumor cells, which generate a channel network facilitating blood supply for tumor growth [54, 55]. Many studies have pointed out that some clinical anti-angiogenic treatments have not been satisfactory, which could be, in part, attributed to VM processes. VM is therefore associated with poor prognosis, reduced survival and high risk of cancer recurrence in addition to resistance to anti-angiogenic treatment [13, 54, 56]. This process has been documented in many different human tumors [57], but to our knowledge has never been reported in normal physiological blood vessel formation. Therefore, in this study we aimed to investigate whether a process closely resembling VM can also occur during the regeneration of normal tissue. In addition, we wanted to identify the main cell type involved in this process. To address this question, the well-described zebrafish fin regeneration assay [34] was employed.

It has been previously described that a functional microcirculation network in malignant tumors is built by three patterns: (i) pre-existing endothelium-dependent blood vessels; (ii) mosaic blood vessels; and (iii) VM channels. Pre-existing endothelium-dependent blood vessels are incorporated into the tumor lesion, mosaic vessels are lined by both tumor cells and ECs, and VM channels contain tumor cells that line channels and mimic endothelial function [12, 58, 59]. Similar microcirculation networks have been documented for the first time in zebrafish caudal fin regeneration at 3dpa containing three vascular structures: normal blood vessels built by well-differentiated ECs, mosaic vessel containing typical EC and Endothelial-Like Cells (ELC), and channels built by ELCs (Fig. 1). This vascular network is fully perfused, as indicated by the presence of red blood cells (RBC) in the lumen. Rounded ELCs extend long sleeves surrounding vessel lumen and morphologically, ELCs closely resemble the macrophage (MΦ) structural phenotype. At the vascular front, multiple extravascular RBCs surrounded by multiple MΦs are revealed (Fig. 1a). Furthermore, we detected perfused mosaic blood vessels built by well-defined ECs and atypical cells appearing as channels in the regenerating fin at 7dpa (Fig. 2). At the channel tip, this cell appears to be a MΦ that makes contacts with the ECs. This cell extends cytoplasmatic extensions and elongates into the blood vessel. Based on this morphological observation, we then examined the MΦ appearance in vivo in the regenerating fin at the 7dpa. MΦs appeared near blood vessels, in the interray fin region, and in the front of the vessel tips (Fig. 2, Video 1). MΦs in front of vessel tips are actively interacting with ECs; making contacts and moving between them (Video 1). It is possible that they are involved in the tissue remodeling process by making channels and providing (drilling) space for the vessel growth. Similar roles of MΦs have been documented in breast cancer, where Obeid and colleagues [60] showed that tumor-associated macrophages (TAMs) could function as channels helping tumor cells to invade the ECM and thus contribute to tumor growth, and metastasis through an angiogenic pathway. Later in 2016, Barnett at al. reported a new role for TAMs forming functional VM channels proximal to cancer stem cells in melanoma [43]. In addition, an association between MΦs and blood vessel sprouting has been shown in vitro and in vivo during embryonic development [42], but MΦs obtaining an ELC phenotype and making perfused channels closely resembling the VM process, to our knowledge, has not been reported in normal regenerating tissue.

During the VM process, aggressive tumor cells can transdifferentiate into multiple cellular phenotypes and obtain endothelial-like characteristics and form channels that mimic blood vessel function [11, 61]. We therefore investigated whether MΦs possess a similar role to tumor cells during VM in the regeneration process of normal tissue. To examine phenotypic changes, flow cytometry analysis and immunofluorescence staining were performed revealing the presence of cells expressing both EC (fli1a) and MΦ (mpeg1) markers, suggesting that these cells are possibly differentiating from one cell type (MΦ) into other (EC) (Fig. 4). Both markers (fli1a and mpeg1) are typically cell line-specific and not co-expressed in the same cell line. It is well know that zebrafish fli1a labels EC and is highly expressed in vascular endothelium [62]. Therefore, the transgenic line is frequently utilized to observe individual migrating ECs [63]. On the other hand, mpeg1 was identified as a gene with expression tightly restricted to macrophages and has subsequently been used as a marker for this cell lineage in zebrafish [47]. According to the flow cytometry study, the percentage of double positive cells over 10 days has a Gaussian distribution in which the peak is reached by 3dpa. Interestingly, the peak is reached at the same time point in which multiple ELC and mosaic vessels were documented (Fig. 1). Afterwards, the amount of double positive cells (in %) decreased. We believe this is the result of cells differentiating into ELCs and adopting an EC phenotype, as documented by morphological observation (Fig. 2). Furthermore, double positive cells were confirmed by IF staining, where EC and MΦ markers were detected within one cell (Fig. 4).

To elucidate whether the process closely resembling VM is present during the regeneration of normal tissue, the VM inhibitor, CVM-1118, was used. Previous studies in vitro have reported that CVM-1118 results in reduced proliferation, induced apoptosis and importantly, reduced formation of branching, tubular networks that are characteristic of VM [10]. Our data, in vivo, demonstrated severe impairments in tissue regeneration and blood vessel formation described by a shorter vascular plexus, less pronounced blood vessels, and the absence of the regeneration process, including blood vessel formation, in half of the fin area (Fig. 5). In the fin area where the regeneration process is present, tissue and vascular plexus appear shorter with dense capillary meshwork containing multiple sprouts, indicating that regeneration is most likely supported by sprouting angiogenesis and not by VM. Interestingly, TRA and VPA are decreased, which means that the fin regeneration and blood vessel formation is impaired (Fig. 5). The reason for the complete absence of half of the regenerating fin could be due to induced apoptosis, reduced proliferation and severe disruption of the formation of vascular networks caused by CVM-1118, which is consistent with previous in vitro studies [10]. In addition, our data showed that CVM-1118 has a cytotoxic effect on MΦs, specifically, significantly reducing their numbers by 60% in comparison with the control animals (Fig. 6). To our knowledge, this effect on MΦs is documented for the first time and further studies involving molecular pathways underlying the interaction between CVM-1118 and MΦs are needed. Taken together, treatment with the vascular mimicry inhibitor (CVM-1118) significantly impairs tissue regeneration by obstructing vessel formation and expansion. Additionally, cytotoxic effect on MΦs has been show.

Further, we decided to investigate the effect of macrophage inhibitor PLX-3397 on caudal fin regeneration, blood vessel formation and MΦ appearance. It is well known that PLX-3397 inhibits the survival, differentiation, and proliferation of MΦs [50, 64, 65]. The role of the PLX-3397, its target, colony-stimulating factor 1 receptor (CSF1R), and impact on macrophages has been mainly investigated in the tumor environment. It is known that CSF1 plays a significant role in the recruitment of peripheral blood monocytes to the TME, differentiation into macrophages, and polarization of macrophages toward an M2-like phenotype via binding to the CSF1 receptor. It has been demonstrated that PLX3397 suppresses survival, migration, and M2 polarization of sarcoma TAMs, and induces their depletion [66–68]. Additionally, it has been shown by Conedera and colleagues in 2019 [50] that PLX3397 treatment significantly diminishes the influence of microglia-macrophages on the injury response. In our study, PLX-3397 was used to reduce the amount of macrophages present after the fin amputation (Fig. 6). Consistent with previous studies, our data showed a significantl reduction in MΦs (about 50%) upon treatment relative to controls. Moreover, the regenerative area and vascular plexus are impaired, capillaries are less prominent and the intraray region is improperly vascularized (Fig. 7), indicating that MΦs play a significant role in supporting proper tissue regeneration and vascularization. Furthermore, In vivo observation are supported by quantification using TRA, VPA and MΦ quantity, showing that upon PLX-3397 treatment all three variables are significantly decreased. Besides well-known importance of MΦs in proper fin regeneration [39, 40], we show the importance of MΦs in proper blood vessel formation during caudal fin regeneration where they contribute to the process closely resembling VM. Along with this, we think that by applying the MΦ inhibitor PLX-3397, we are reducing the amount of MΦs, which will subsequently transform in ELCs and help to form functional blood vessels.

The relationship between MΦs and tissue regeneration has been well studied and documented, as MΦs were shown to be essential during wound healing, tissue repair and tissue remodeling [69, 70]. MΦs display a high-functional plasticity with respect to shape, behavior changes during the immune response, gene expression, and supporting sprouting angiogenesis by bridging the gap between ECs in damaged endothelium and secreting some angiogenic growth factors [41, 42, 71, 72]. Here, we have revealed an additional characteristic of MΦs; demonstrating their capacity to differentiate into ELCs, adopt EC morphological features and contribute to neoangiogenesis by VM in the regenerating tissue. This opens new questions and gives a fresh perspective on the VM process.

In the present study, we are able to show that a process closely resembling VM, is present as well during the regeneration of normal, healthy tissue. During the early tissue regeneration process, vasculature is built by the mosaic blood vessels containing both ECs and ELCs. ELCs appear, morphologically, like MΦs and express both EC and MΦ markers (detected as double positive cells). Those cells appear at the regenerating front, adopt an EC phenotype and form functional and perfused channels. The vasculature is therefore partially expanded by transformation of the adjacent MΦ into ELCs. The mechanisms behind this process, however, still require further investigation. Overall, our study opens new perspectives on the physiological role of VM contributing to blood vessel regrowth during tissue regeneration and opens a window for innovative therapeutic options.

### Electronic supplementary material

Below is the link to the electronic supplementary material.

Supplementary Material 1

Supplementary Material 2

## Data Availability

All data generated or analyzed in this study are included in this article.
