# Supplementary material for: Vascular mimicry in zebrafish fin regeneration: how macrophages build new blood vessels
Source: Angiogenesis. 2024 Mar 28;27(3):397–410. doi: 10.1007/s10456-024-09914-y (PMC11303510; doi:10.1007/s10456-024-09914-y)
Supplement: Supplementary file 2 — Supplementary Material 2 [file 10456_2024_9914_MOESM2_ESM.docx]

**Video 1 The vascular segments are expanding following the macrophages at the tips.**
